# Phalangeal joints kinematics in ostrich (*Struthio camelus*) locomotion on sand

**DOI:** 10.1371/journal.pone.0191986

**Published:** 2018-02-28

**Authors:** Rui Zhang, Qiaoli Ji, Dianlei Han, Haijin Wan, Xiujuan Li, Gang Luo, Shuliang Xue, Songsong Ma, Mingming Yang, Jianqiao Li

**Affiliations:** Key Laboratory of Bionic Engineering, Ministry of Education, Jilin University, Changchun, P.R. China; Brown University, UNITED STATES

## Abstract

In ostriches, the toes are the only body parts that contact loose sand surfaces. Thus, toe interphalangeal joint motions may play vital biomechanical roles. However, there is little research on ostrich phalangeal joint movements while walking or running on sand. The results from the three-dimensional motion track analysis system Simi Motion show that gait pattern has no significant effect on the key indicators (angles at touch-down, mid-stance, lift-off and range of motion) of the toe joint angles. The motion of the toe phalanges when walking and running on sand is basically the same. The ground medium is the key factor that changes the toe postures adopted by ostriches during the stance phase in slow to fast locomotion. The 3rd toe and the 4th toe are connected by the interphalangeal ligament, and the motions of the MTP3 and MTP4 joints are highly synchronized on a loose sand substrate. The 3rd toe and 4th toe are coupled to maintain static balance in slow locomotion and dynamic balance in fast locomotion. In addition, the gait pattern has a marked effect on the range of forward displacement of the toenail (*Y*_*TN*_). The ostrich toenail plays an important role in preventing slip and provides traction at push-off in a sandy environment. The metatarsophalangeal joint plays an important role in energy saving during fast locomotion on loose sand substrates. Simulation reveals that the particle velocity field, particle force field and sand particle disturbance in the running gait are denser than those in the walking gait.

## Introduction

The ostrich (*Struthio camelus*) is acknowledged as the fastest and largest extant bipedal terrestrial animal with remarkable speed and exceptional endurance during locomotion on sandy environment and wasteland [1–4]. The ostrich can run for 30 minutes at 50 km/h, moving at a speed of 70 km/h for short sprints. It has a remarkable combination of speed and endurance. Interestingly, compared with other birds, the heavy and large ostrich has only two toes, namely the 3rd major claw-bearing toe and the lateral 4th toe [2,4]. In addition, the supra-jointed toe posture is the unique adaptation of the ostrich. This is because the metatarsophalangeal joint and the proximal phalanx of both toes are permanently elevated above the ground plane [5]. Therefore, the ostrich only relies on the two digits from slow walking to fast running in locomotion and is truly digitigrade [4]. Previous studies described the ostrich hindlimb morphology, toe function dynamic pressure distribution and phalangeal joint kinematics during walking and running gaits, which provided the research basis for the unique locomotor performance on solid ground [4, 6–8, 9].

Some studies show that the 3rd toe of the ostrich has four phalanges, and the toenail and the 4th toe has five phalanges [10–11]. Phalangeal joint kinematics of ostriches on solid surfaces have been studied [9]. However, ostriches often live in natural environments with loose sand surfaces, and toe interphalangeal joint kinematics on loose sand substrates have not been quantitatively measured. Therefore, it is important to explore the interphalangeal joint kinematics of toes and the interaction between ostrich toe plantar surface and sand particles, which may help us to understand the biomechanical mechanism of ostrich toes to travel on sand and study the special running performance of ostriches.

Many animals run on soft soil and loose sand terrain in natural environments. Thus, some researchers have studied the locomotion of some animals on deformable surfaces. For example, the high locomotor performance of the zebra-tailed lizard on surfaces and flowing substrates under stress has been studied, and a penetration force model substrate was provided for studying locomotion on flowing substrates [12–13]. The research showed that the hind foot of the zebra-tailed lizard lost energy per step during irreversible deformation of the substrate while running at ~1 m s^-1^ on the granular surface [12–13]. In addition, three-dimensional foot movements of guinea fowl that traverse a granular subsurface have been reconstructed and simulated to provide a dynamic glimpse into the previously invisible process of track formation, called “track ontogeny” [14]. However, the motion of ostrich toe interphalangeal joints on loose sand substrates has received little attention. To accurately study the hindlimb locomotion mechanism under deformable substrate, it is necessary to collect phalangeal joint kinematics data of an animal’s foot on a loose sand surface.

In previous work, we studied the relative motion of interphalangeal joints of ostrich toes on solid ground. However, how do the ostrich toe phalanges move on sand substrate?

Why can the heavy and large ostrich maintain such a high speed in a sandy environment? We have been very interested to answer these questions. Therefore, in this paper, we explored the phalangeal joint kinematics and displacement of the toenail of ostrich toes during the stance period in *vivo* on a loose sand surface to analyze the specific role played by the toes in providing support, grip and balance in walking and running gaits.

Additionally, we constructed five ostrich toe postures and simulated the process of traveling on sand during ostrich locomotion using EDEM^®^ 2.7 software (Edinburgh, UK). Thus, our primary aims were to test the following three questions. Firstly, on loose sand substrates, how do ostrich toe interphalangeal joint angles change with gaits from walking and fast running during the stance phase? Secondly, are there different effects of gait pattern and ground medium on the phalangeal joints and displacement of the metatarsophalangeal joint (in the vertical, forward and lateral directions) and toenail (in the forward and lateral directions) during the stance period?

Finally, are there differences in sand particle locomotion under the ostrich toe plantar surface between walking and running? To test our aims, phalangeal joint angles and the vertical displacement of the metatarsophalangeal joint at touch-down, mid-stance, lift-off, joint range of motion, maximum and minimum were selected as key indicators for statistical analysis. In addition, the range of motion of displacement in the forward and lateral directions of the metatarsophalangeal joint and toenail were also selected as key indicators for statistical tests. We also simulated the process of traveling on sand and analyzed the velocity and force field of sand particles during walking and running gaits.

We acquired the kinematics data of the interphalangeal joints of ostrich toes on a loose sand surface during the entire stance phase using a three-dimensional motion analysis system (Simi Motion 2D/3D^®^ 7.5 software, SIMI Reality Motion Systems GmbH, Germany). Statistical analysis was also conducted to investigate the effect of gait pattern and ground medium on interphalangeal joint motions and the displacement of the metatarsophalangeal joint and toenail. This study may further promote our understanding of the in *vivo* biomechanical function of ostrich toes and their contribution to the overall locomotor performance of ostriches on loose sand surfaces. Finally, the theoretical study may provide new ideas and inspirations for the joint design of walking legs for bionic walking robots in sandy environments.

## Materials and methods

### Animals

Ten healthy sub-adult ostriches (*Struthio camelus*) with an average age of eight months were selected from the Ji’an breeder, Jilin province, P.R. China. The two tractable female ostriches were selected as objects to complete all tests. The average mass and height of the two ostriches are 83.5 ± 2.02 kg and 2.10 ± 0.02 m (displayed by means ± S.D.), respectively. Without any form of surgical treatment or invasive physical manipulation, the individuals were in excellent physical condition with the properly elevated metatarsophalangeal joints, which represented the average body proportion and weight for ostriches of their age and sex [5]. These two ostriches were kept in an outdoor enclosure during daytime with unlimited access to food and water and housed in an indoor enclosure during nighttime. Each bird was trained to walk and run in a fenced-in corridor for at least 30 minutes per session and twice per day over a month before data collection. After comprehensive comparison of representation and amenability, the two ostriches finished the locomotion experiments of toe phalangeal joints. All living and experimental conditions were approved by the Institutional Animal Care and Use Committee (IACUC, protocol number: 20140706) of Jilin University, P.R. China.

### Surface treatments and trials

The runway was covered with loose sand particles at a depth of 4 cm (diameter = 0.3~1.6 mm, density = 1.6×10^3^ kg·m^-3^). If the sand depth was greater than 4 cm, ostrich toes may be buried in sand, and the marker motion could not be acquired. However, if the sand depth was smaller than 4 cm, the sand substrate had little interaction with ostrich toes, and the experimental results may be influenced. Thus, we chose the depth of the sand as 4 cm. Before each trial, the sand surface was leveled [9]. Experimenters randomly varied the speed from slow walking to running across trials and allowed ample rest and food between trials to prevent fatigue. Experiments were canceled if animals showed fatigue that would cause discomfort or adversely affect our measurements. To minimize the interference of sunlight, one sunshade net was set on the top of the data acquisition zone.

### Phalangeal joint angles and displacements

**Fig 1:**
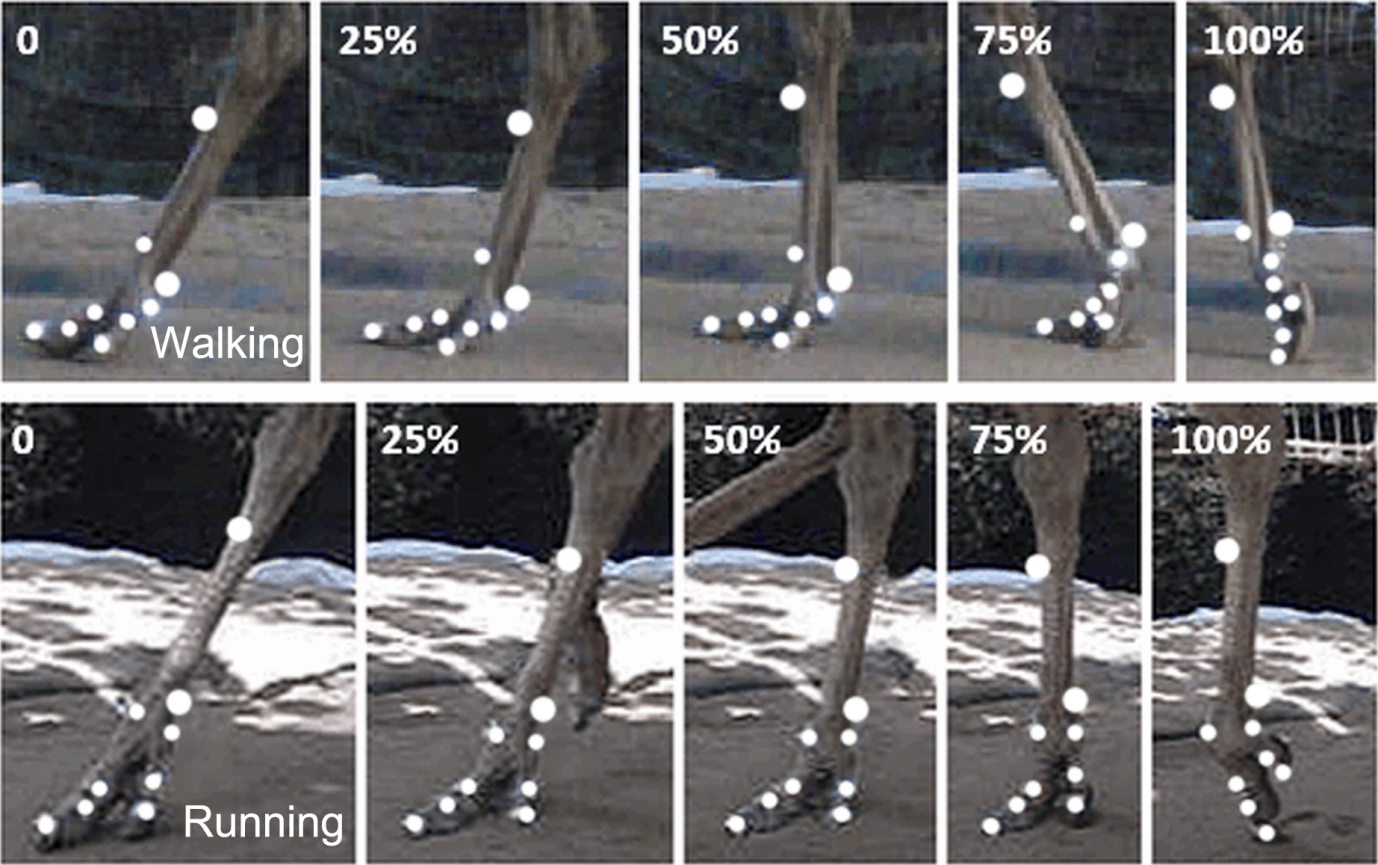
On loose sand substrate, two representative high-speed video traces of toe motion during slow walking and running in the stance phase. The traces started at touch-down when the 3rd toe touched the ground at 0% of the stance phase. In the slow walking and running trials, the mid-stance is at 50% of the stance phase, and the 3rd toe cleared off the ground at 100% of the stance phase.

We used three synchronized high-speed cameras (Casio Exilim EX-FH25, Tokyo, Japan) to measure the 3D coordinates of the nine retro-reflective markers at 240 frames s^-1^. Then, we used a three-dimensional motion tracking system (Simi Motion 2D/3D^®^ 7.5 software, SIMI Reality Motion Systems GmbH, Germany) to capture the kinematics data of six joint angles (*α*, *β*, *γ*, *θ*, *φ*, *ψ*), vertical displacement of the metatarsophalangeal joint (*z*) [9], angle (*α*) between phalanges II and III of the 3rd toe, angle (*β*) between phalanges I and II of the 3rd toe, angle (*γ*) between tarsometatarsus and phalanx I of the 3rd toe (MTP3 joint), angle (*θ*) between tarsometatarsus and phalanx I of the 4th toe (MTP4 joint), angle (*φ*) between phalanges I and II of the 4th toe, angle (*ψ*) between the first phalanges of the 3rd and 4th toes and all angles were spatial 3D angles. Displacement of the metatarsophalangeal joint (*Y*_*MTP*_, *X*_*MTP*_) was in the forward and lateral directions, and displacement of the toenail (*Y*_*TN*_, *X*_*TN*_) was in the forward and lateral directions. Two series of representative video frames recorded for slow walking and running, respectively, are shown in Fig 1.

Gait parameters, including cycle period, stance duration, swing duration, duty factor and stride length, are listed in Table 1. In this study, trials with velocities in the range od 0.88~1.70 m s^-1^ with a stance phase duration of 0.56~1.08 s and duty factor above 0.5 were identified as walking gaits at slower speeds with double support, whereas trials velocities in the range of 2.66~4.09 m s^-1^ with a stance phase duration of 0.27~0.37 s and duty factor below 0.5 were regarded as running gaits [4]. There were 22 valid trials, of which 13 were walking trials and 9 were running trials, which are shown in Table 1.

**Table 1:** The key gait parameters during slow walking and running on loose sand substrate.

Gait parameters	slow walking (0.88−1.70 m/s)	running (2.66−4.09 m/s)
Number of trials	13	9
Average speed (m/s)	1.19±0.35	3.21±0.55
Froude numbers	0.32±0.10	0.87±0.15
Duty factor	0.62±0.11	0.45±0.05
Stance phase (second)	0.82±0.26*	0.32±0.05*
Swing phase (second)	0.46±0.12	0.40±0.02
Cycle period (second)	1.28±0.26*	0.72±0.03*
Stride length (meter)	1.44±0.12*	2.29±0.29*

Note that values are means ± S.D.

*Statistically significant gait effects are indicated by an asterisk (P<0.05).

### Statistical analysis

We examined the differences of four gait parameters (stance and swing duration, cycle period and stride length) and six key indicators (phalangeal joint angle/the vertical displacement of the metatarsophalangeal joint at touch-down, mid-stance, lift-off, range of motion, maximum and minimum, the displacements of the metatarsophalangeal joint and toenail in the forward and lateral directions at the range of motion) between slow walking and running gaits. Additionally, we tested the effect of ground medium on the six key indicators of phalangeal joint angles between solid ground and loose sand surfaces. We used a one-way ANOVA to analyze the effect of gait pattern and the ground medium on each gait parameter or joint angle/displacement indicator [15–16]. We used an F-test to test whether these two variations were significantly different, and statistical significance level was considered as P<0.05. To study the potential for inter-subject variation, interphalangeal joint angle values and displacements of slow walking and running trials from each individual were analyzed using an ANOVA. A total of 22 samples (individual A, 11 samples; individual B, 11 samples, see Table 2), divided between slow walking and running trials, were included in the statistical analysis. The statistical analysis number for walking and running trials were equal. In addition, we ran an ANOVA to test the effect of substrate (solid surface and loose sand surface) on the joint angle/displacement indicator in the same gait pattern (slow walking and running gaits). All statistical tests were performed using Origin Pro 2015 software (OriginLab Corporation, Northampton, USA).

**Table 2:** The statistical analysis trials number of two individuals during walking and running gaits.

	Walking trials		Running trials	
Individuals	A	B	A	B
Valid trials	6	7	5	4
Statistical analysis trials	6	6	4	4

### Three-dimensional modeling of ostrich toe postures

**Fig 2:**
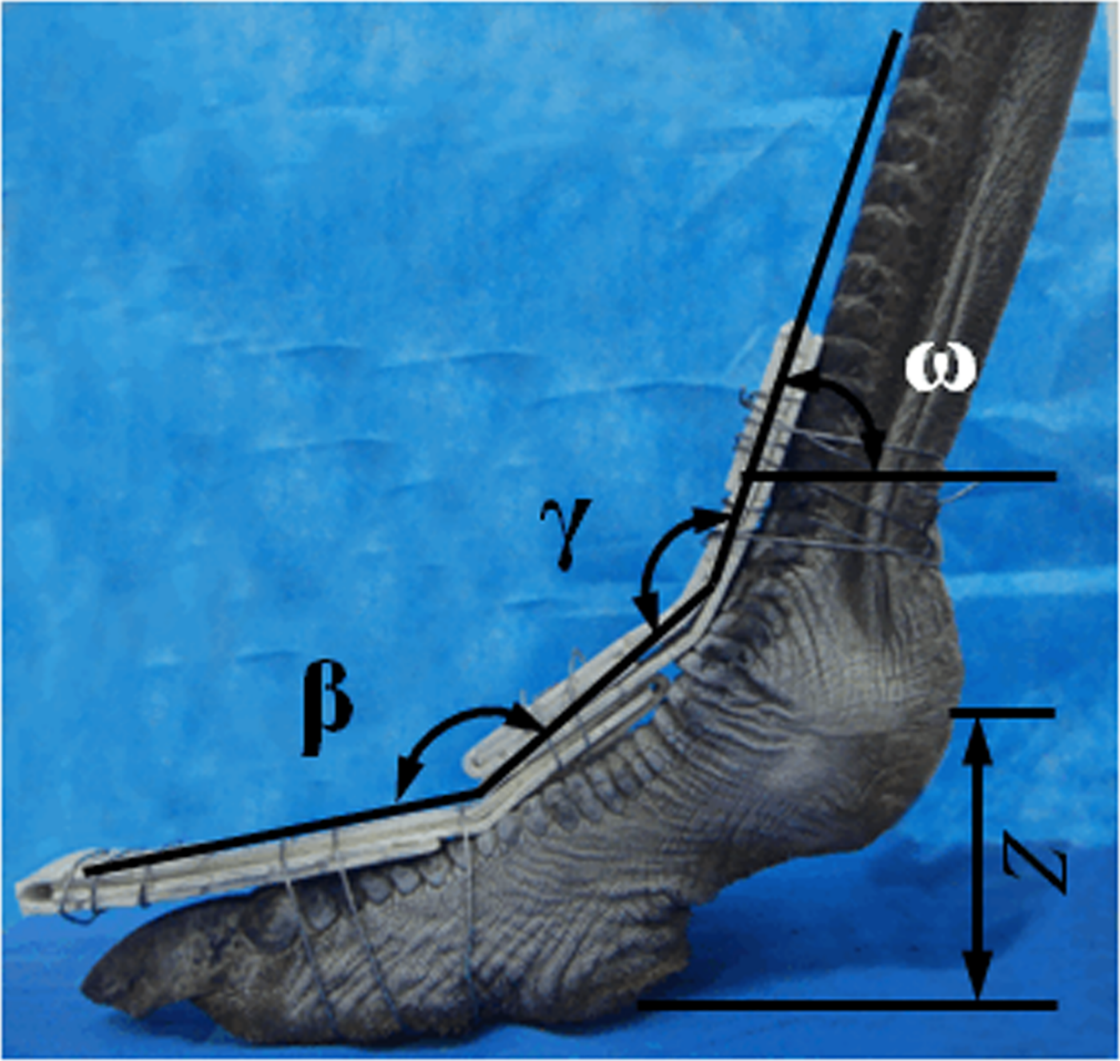
The parameters of ostrich toe fixed posture. Angle *β* between phalanges I and II of the 3rd toe, angle *γ* between the tarsometatarsus and phalanx I of the 3rd toe (MTP3 joint), *Z* the vertical displacement of the metatarsophalangeal joint, angel *ω* between the tarsometatarsus and the horizontal plane.

**Fig 3:**
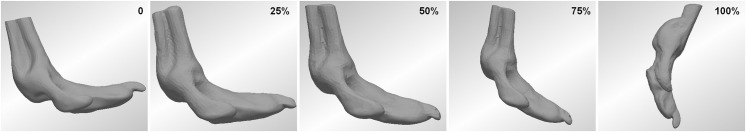
Five ostrich toe posture models. At touch-down (0 posture), mid-stance (50% posture), lift-off (100% posture), 25% and 75% of the stance phase corresponding to 25% posture and 75% posture.

After the process of acquiring phalangeal joint kinematics data, we collected and analyzed the key parameters of ostrich toe posture. According to the four parameters of phalangeal joint angle (*β*) between the first phalanx and the second phalanx of the 3rd toe, the phalangeal joint angle (*γ*) between the first phalanx of the 3rd toe and the tarsometatarsus, angel (*ω*) between the tarsometatarsus and the horizontal plane, the vertical displacement (*Z*) of the metatarsophalangeal joint (see Table 3), we confirmed five ostrich toe postures that corresponded to five stance moments (at 0, 25%, 50%, 75%, 100% of the stance phase). At first, the thermoplastic plate was fixed as a fixture of the ostrich toe posture (at 0 of the stance phase) and ensured the ground height of the metatarsophalangeal joint and the fixed posture, as shown in Fig 2. Then, the three-dimensional laser scanner was used to acquire the point cloud data of the ostrich toe posture. Finally, point cloud data of the ostrich toes were input to the Reserve Engineering Software (Geomagic Studio^®^, Geomagic Corporation, North Carolina, USA), and we obtained a 0 posture model of ostrich toe posture. Finally, we used a similar method to obtain different toe postures during the stance phase, namely, 25% posture, 50% posture, 75% posture, and 100% posture (see Fig 3).

**Table 3:** Interphalangeal joint angle and displacement parameters of ostrich toes.

Stance Moment /%	*β*/°	*γ*/°	*ω*/°	*Z*/cm
0	158±14	165±8	54±5	12.8±2.6
25	144±13	159±15	62±4	6.6±2.4
50	138±15	154±16	75±4	7.0±1.1
75	134±20	163±15	79±3	14.6±0.9
100	156±10	186±15	80±3	24.6±1.9

Note that values are displayed as means ± S.D.

### Using EDEM to simulate the process of ostrich toes traveling on sand

The numerical simulation software (EDEM^®^ 2.7.0 software, DEM Solutions Corporation, UK) was used to simulate the loose sand substrate. Validation simulations were carried out to determine the material properties required for simulating the sand particles. By combining experiments and simulations of the angle of repose of sand particles [17], we acquired the parameters of simulated sand particles and then constructed a 700 mm long, 240 mm wide and 180 mm deep virtual sediment box to simulate the sand ground. The simulated sand particle had a uniform size with a 1.0 mm radius. Sand particles were created in the virtual box and allowed to settle under gravity until at rest. The height of the sand particles was approximately 60 mm. To avoid major boundary effects, the sediment volume was sufficiently large to encompass all significant particle displacements.

Because actual ostrich toes exhibit relative motion of the interphalangeal joints in locomotion, the ostrich toe postures (0 posture, 25% posture, 50% posture, 75% posture, 100% posture) were still rigid models. Therefore, to simplify the simulation process of traveling on sand, the velocity and acceleration of point C between phalanges I and II of the 3rd toe were considered the motion pattern of five ostrich toe postures [9]. Then, we exported the velocities and accelerations of point C during the stance phase in the walking and running gaits on the loose sand surface using the motion capture system Simi Motion. The velocities and accelerations of 0 posture, 25% posture, 50% posture, 75% posture, and 100% posture corresponded to stages 0~20%, 20%~40%, 40%~60%, 60%~80%, and 80%~100% of the stance phase, respectively. The motion of five ostrich toe postures was set to the discrete element simulation (see S1 Table). In the simulation process, all sand particle velocities and displacements were recorded at each time step.

In addition, it was important to note that the toe posture models in the simulation have no mass, and we only focused on the effect of toe posture on sand particles. Different toe postures correspond to different time periods of the stance phase. The entire simulation process of ostrich toes traveling on sand was composed of five small stages, which were the motion (0~20% of the stance phase) of 0 posture, the motion (20~40% of the stance phase) of 25% posture, the motion (40~60% of the stance phase) of 50% posture, the motion (60~80% of the stance phase) of 75% posture, the motion (80~100% of the stance phase) of 100% posture.

## Results

### Gait parameters

Table 1 shows the averages and standard deviations of stance duration, swing duration, cycle period, and stride length of all walking and running gaits on loose sand substrate. There were statistically remarkable differences in stance duration, cycle period and stride length, which showed that ostriches used considerably shorter cycle periods and stance duration during running than walking, whereas they significantly increased their stride lengths. This is consistent with previous observations [2]. No statistically significant difference was found in swing duration between the walking and running gaits. These observations were consistent with the gait parameters of ostrich locomotion on solid ground [9].

### Toe joint kinematics on loose sand substrate

**Fig 4:**
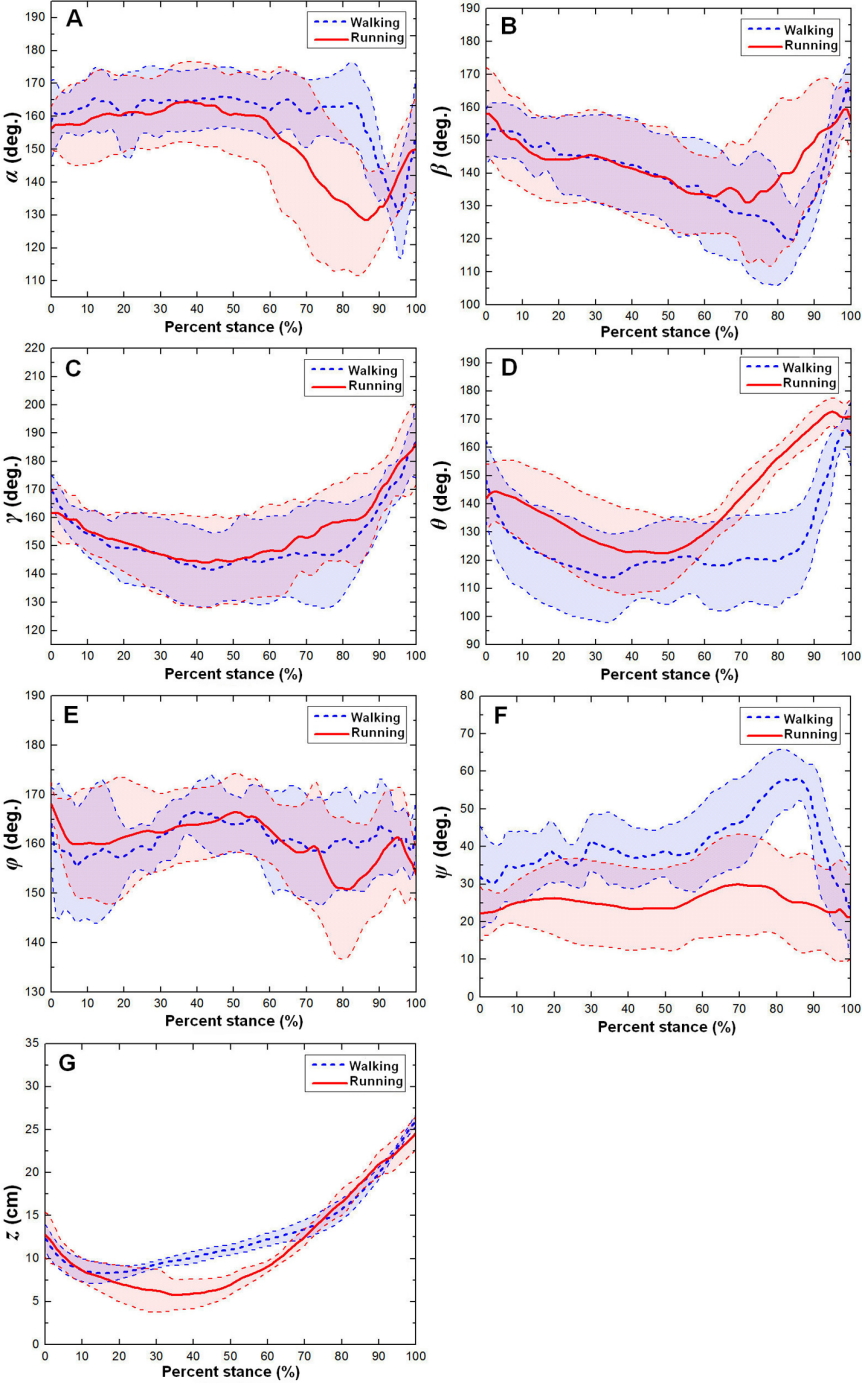
The averages and one standard deviations of the six toe joint angles and the vertical displacement of the metatarsophalangeal joint (*α*, *β*, *γ*, *θ*, *φ*, *ψ*, *z*). They correspond to A B C D E F G, respectively, over the stance phases for all slow walking (blue dotted line) and running trials (red solid line) on loose sand substrate. The angle decrease represents flexion, while the angle increase indicates extension.

Fig 4 and S4 Table show on a loose sand substrate, the averages and standard deviations of the six toe joint angles and the vertical displacement of the metatarsophalangeal joint (*α*, *β*, *γ*, *θ*, *φ*, *ψ*, *z*) during the stance periods for walking and running trials. The time trajectories of the first phalangeal joint angle of the 3rd toe (*α*)represented similar motion patterns at the stance phases in the walking and running gaits. In slow walking, the first phalangeal joint of the 3rd toe remained at approximately 162 degrees throughout from the touch-down to late stance (at 85% of the stance phase). Then, up to lift-off, it revealed a quick flexion of approximately 30 degrees and a rapid extension of approximately 25 degrees. However, there was a difference between the walking and running gaits, which represented the joint remaining stable from the early stance to the mid-stance during running (from touch-down to 60% of the stance phase); thereafter, the joint angle decreased from 160 degrees to 130 degrees (from mid-stance to 90% of the stance phase). Then, the joint extended approximately 20 degrees in the late stance earlier than that of walking (see Fig 4A). As shown in Fig 4B, the second phalangeal joint angle of the 3rd toe (*β*) displayed almost coincident motion patterns from early stance to mid-stance (at 60% of the stance phase) of walking and running. In walking, the joint angle (*β*) decreased after touch-down from approximately 150 degrees to 120 degrees at late stance. After that, the joint extended quickly back to approximately 166 degrees before the lift-off stage. In the running gait, the joint angle (*β*) decreased after touch-down from approxi1mately 60 degrees to 130 degrees (at 70% of the stance phase) and extended rapidly back to approximately 160 degrees just before lift-off.

The MTP3 joint angle (*γ*) presented similar patterns (from touch-down to 40% of the stance phase) and different patterns (from touch-down to 60% of the stance phase) during walking from running (see Fig 4C). In walking, the joint angle (*γ*) decreased gradually by approximately 15 degrees from touch-down to 40% of stance phase and then remained stable at 145 degrees until reaching 80% of the stance period. A quick joint angle increase occurred before lift-off with the MTP3 joint extended approximately 40 degrees. However, there was no steady stage in the middle stance in running. The MTP3 joint flexed approximately 15 degrees progressively from touch-down to middle stance and extended increasingly to 186 degrees.

As shown in Fig 4D, the MTP4 joint angle (*θ*) reveled noticeably different motion patterns between walking and running. After touch-down, the MTP4 joint flexed approximately 35 degrees quickly and kept stationary from early stance (at 30% of the stance phase) to late stance (at 80% of the stance phase) in walking. Thereafter, a quick joint extension occurred at the MTP4 joint, reaching an extended position at 165 degrees just before lift-off. However, there was not a stationary period at the middle of stance phase in running. The MTP4 joint flexed approximately 20 degrees from touch-down to middle stance and then showed an increasing joint extension of 50 degrees before lift-off.

The first phalangeal joint of the 4th toe (*φ*) displayed the largest angle variability among all six toe joints, as shown in Fig 4E. The joint angle (*φ*) did not present significant motion patterns in walking and running. The joint angle (*φ*) fluctuated approximately 165 degrees, although it revealed that greater variability occurred during both walking and running. However, as shown in Fig 4F, the joint angle (*ψ*) between the first phalanges of the 3rd and 4th toes displayed obvious patterns during the stance period in the walking and running gaits. The angle (*ψ*) displayed a gradual abduction (30 degrees) increasing between the 4th toe and the main axis of the 3rd toe from touch-down to late stance (at 80% of the stance phase) and then showed a quick adduction (35 degrees) before lift-off during the walking gait. In addition, the average peak joint extension was approximately 58 degrees for the walking gait, which was larger than the previous study in which the maximum range of motion angle between the main axes of the 3rd and 4th toes was 34 degrees [4]. Compared to the walking gait, the joint angle (*ψ*) between the two toes remained a stable motion pattern and slightly fluctuated approximately 25 degrees during running (see Fig 4F).

The average and one standard deviation of the vertical displacement of the metatarsophalangeal joint (*z*) during the entire stance periods in walking and running trials are shown in Fig 4G. We found that different motion patterns were displayed between walking and running. Just after touch-down (at 10% of the stance phase), the metatarsophalangeal joint moved downwards towards the ground surface at approximately 3.6 cm and then went smoothly upwards at approximately 16 cm before lift-off in walking. However, the metatarsophalangeal joint only moved downwards slightly at approximately 7.0 cm from touch-down to close to middle stance and thereafter remained going upwards before lift-off at approximately 20 cm in running.

**Fig 5:**
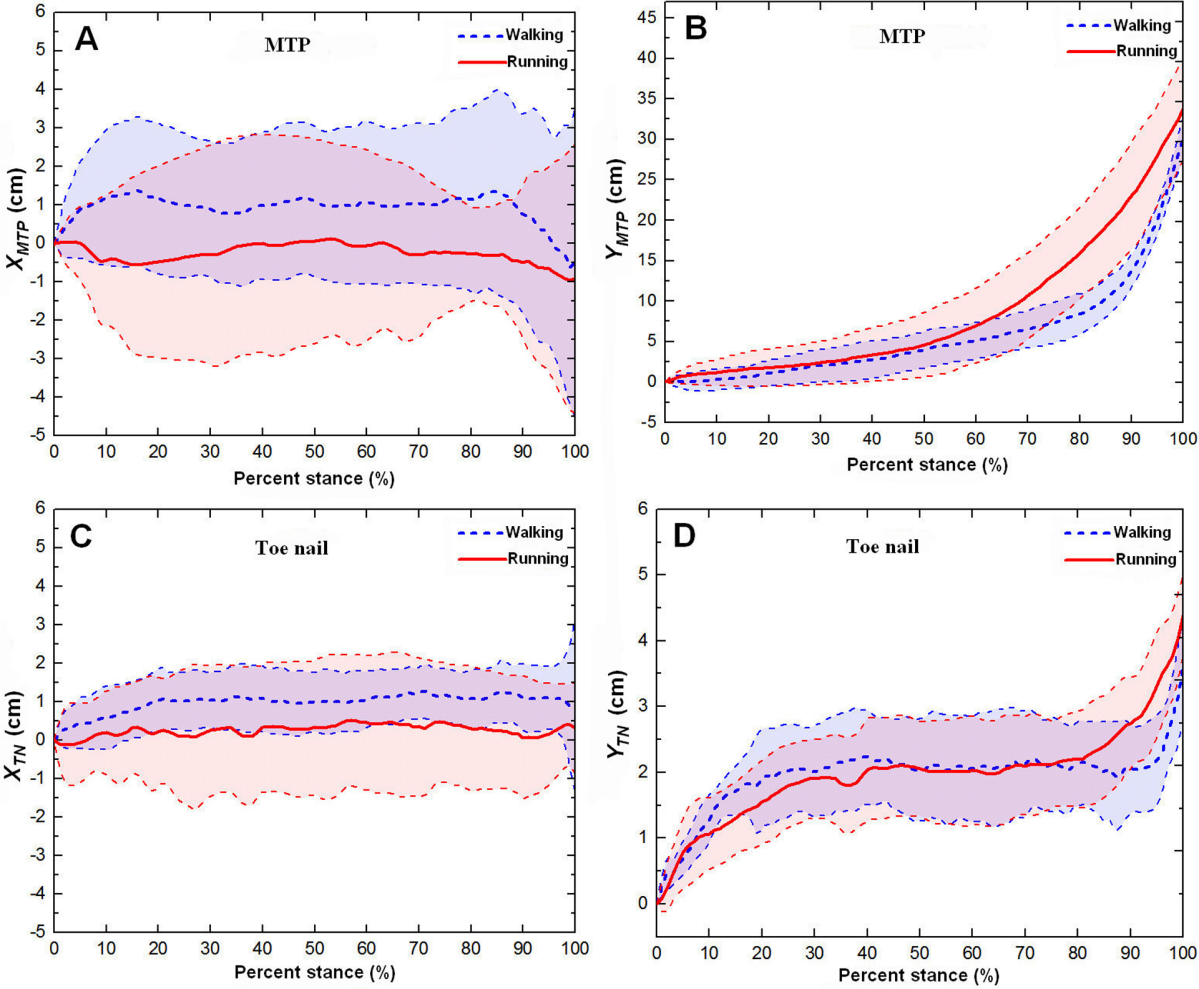
The averages and one standard deviations of the lateral (*X-direction*) and forward (*Y-direction*) displacements of the metatarsophalangeal joint and toenail. *X*_*MTP*_, *Y*_*MTP*_, *X*_*TN*_, and *Y*_*TN*_ correspond to A B C D, respectively, over the stance phases for all walking (blue dotted line) and running trials (red solid line) on loose sand substrate. In the axis of ordinates, positive numbers indicate the movement direction of the metatarsophalangeal joint toward the outside of ostrich feet, and negative numbers indicate the movement direction of the metatarsophalangeal joint toward the inside of the ostrich foot.

Fig 5 and S4 Table show the averages and one standard deviations of the lateral and forward displacements of the metatarsophalangeal joint and toenail (*X*_*MTP*_, *Y*_*MTP*_, *X*_*TN*_, *Y*_*TN*_) on the loose sand substrate over the stance phases for all walking and running trials. From Fig 5A, it can be observed that in walking, the lateral displacement (*X*_*MTP*_) of the metatarsophalangeal joint increased by approximately 1.5 cm from the touch-down to 20% of the stance phase and then remained at approximately 1.0 cm throughout the early stance to 90% of the stance phase. This was followed by a decrease of approximately 2 cm just before lift-off. From Fig 5C, it can be observed that in walking, the lateral displacement (*X*_*TN*_) of the toenail increased by approximately 1.0 cm from the touch-down to 20% of the stance phase and then remained stable before lift-off. However, in the running gait, the lateral displacement (*X*_*MTP*_) of the metatarsophalangeal joint and the lateral displacement (*X*_*TN*_) of the toenail presented no apparent pattern.

Fig 5B shows that in walking and running, the forward displacements (*Y*_*MTP*_) of the metatarsophalangeal joint presented similar motion patterns. In walking, the metatarsophalangeal joint moved at a uniform speed from touch-down to 85% of the stance phase, and the corresponding forward displacement *Y*_*MTP*_ increased by approximately 10 cm. Thereafter, the metatarsophalangeal joint moved quickly along the forward direction, and the corresponding forward displacement *Y*_*MTP*_ increased by approximately 20 cm before lift-off. In running, the metatarsophalangeal joint moved at a uniform speed from touch-down to middle stance (at 50% of the stance phase), and the corresponding forward displacement *Y*_*MTP*_ increased by approximately 5 cm. Then, the metatarsophalangeal joint moved quickly along the forward direction, and the corresponding forward displacement *Y*_*MTP*_ increased by approximately 30 cm before lift-off. The forward displacements (*Y*_*TN*_) of the toenail presented clear motion patterns in walking and running (see Fig 5D.), which first increased and then remained stable and then increased again before lift-off. The forward displacement *Y*_*TN*_ of the toenail increased 2 cm from early stance (from touch-down to 30% of the stance phase) and then remained stable at the middle stance stage. However, in running (at 80% of the stance phase), an increase of the forward displacement *Y*_*TN*_ of the toenail occurred earlier than that in walking (at 95% of the stance phase). In the walking and running gaits, the forward displacements *Y*_*TN*_ of the toenail increased by approximately 1.5 cm and 2.5 cm before lift-off, respectively.

### Effect of gait pattern on key indicators of toe joint angles and displacements

**Fig 6:**
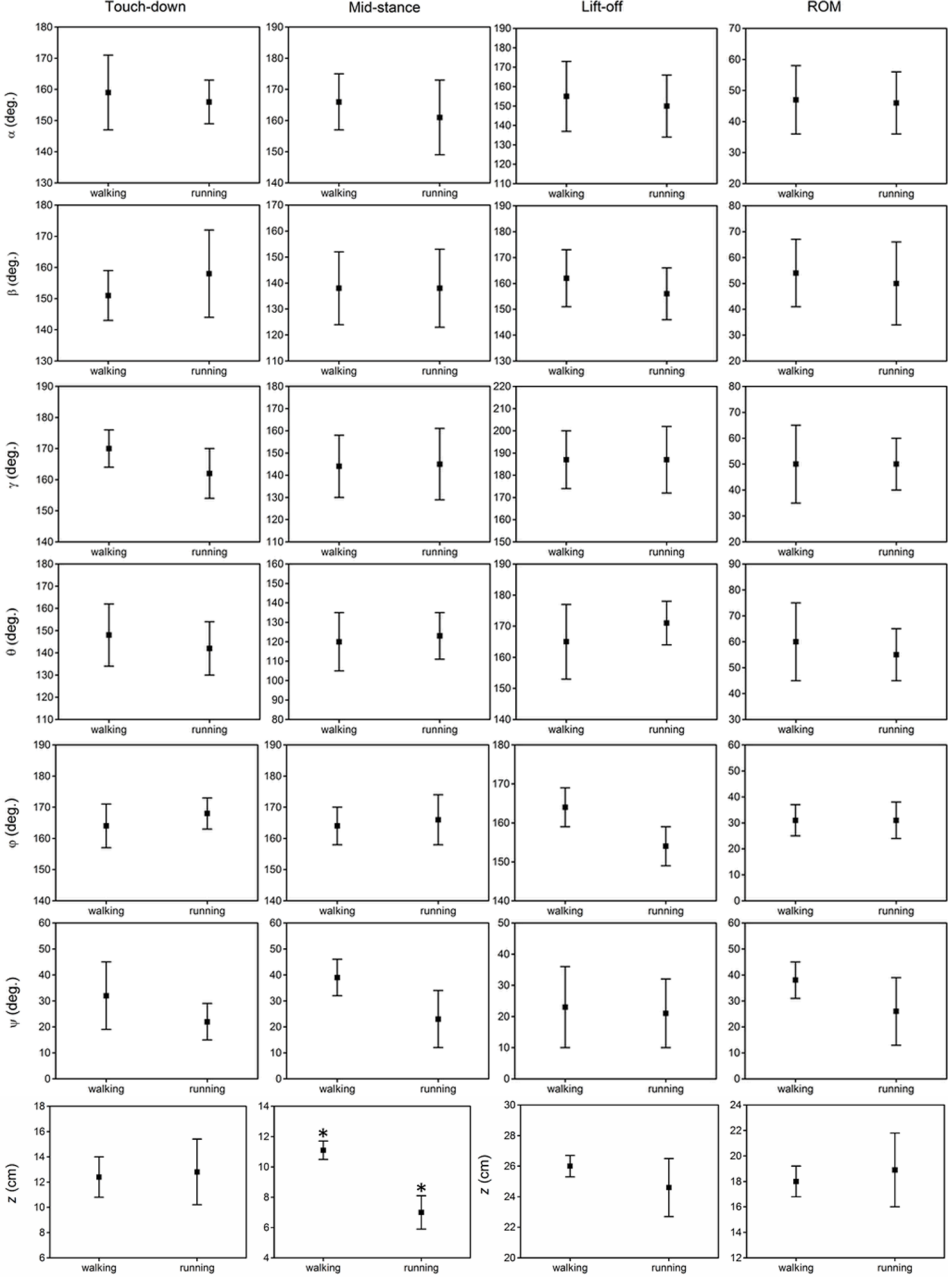
The averages and standard deviations of the six toe joint angles (*α*, *β*, *γ*, *θ*, *φ*, *ψ*) and the vertical displacement of the metatarsophalangeal joint (*z*) at touch-down, mid-stance, lift-off and the ranges of motion during walking and running on loose sand substrate. A statistically significant effect of gait pattern is indicated by an asterisk (P < 0.05).

The results of the statistical analysis testing the effect of gait pattern on the six key indicators (angles/displacements at touch-down, mid-stance, lift-off, maximum, minimum and range of motion) of the six toe joint angles and the vertical displacement of the metatarsophalangeal joint on loose sand substrate are listed in Fig 6 and Table 4. Gait pattern had no significant effect on the key indicators (angles at touch-down, mid-stance, lift-off and range of motion) of the toe joint angles (*α*, *β*, *γ*, *θ*, *φ*, *ψ*). The MTP3 joint angle (*γ*) and the MTP4 joint angle (*θ*) presented very similar motion patterns between walking and running on loose sand. However, statistically significant differences were found for the maximum angle between the first phalanges of the 3rd and 4th toes (*ψ*). The maximum joint angle (*ψ*) was greater during walking than during running and was approximately 60 degrees and 37 degrees, respectively. In walking, the maximum joint angle (*ψ*) was greater than the maximum angle (34 degrees) recorded by a fresh anatomical dissection in previous research. This may suggest that ostrich body stabilization and balance require fine adjustment of the joint angle between the first phalanges of the 3rd and 4th toe, especially for slow locomotion [4]. In addition, statistically significant differences were also found for the mid-stance vertical displacement of the metatarsophalangeal joint (*z*). The heights of the metatarsophalangeal joint at mid-stance in walking and running were 11.1 cm and 7.0 cm, respectively. Therefore, compared to running, the metatarsophalangeal joint was at a statistically higher position at mid-stance during walking, which was consistent with the viewpoints that on solid ground, the metatarsophalangeal joint was positioned closer to the ground as speed increased [3, 9].

**Table 4:** Key indicators of phalangeal joint angles and displacement during walking and running gaits on loose sand substrate.

Parameters	touch-down		mid-stance		lift-off		maximum		minimum		rom	
	walking	running	walking	running	walking	running	walking	running	walking	running	walking	running
*α*	159±12	156±7	166±9	161±12	155±18	150±16	173±5	169±9	126±14	123±16	47±11	46±10
*β*	151±8	158±14	138±14	138±15	162±11	156±10	168±8	169±4	115±11	119±16	54±13	50±16
*γ*	170±6	162±8	144±14	145±16	187±13	187±15	188±12	187±14	138±14	137±12	50±15	50±10
*θ*	148±14	142±12	120±15	123±12	165±12	171±7	170±6	175±4	111±16	120±11	60±15	55±10
*φ*	164±7	167±5	164±6	166±8	164±5	154±5	175±4	173±4	144±6	142±5	31±6	31±7
*ψ*	31±13	22±7	39±7	23±11	23±13	21±11	60±6*	37±12*	22±12	11±5	38±7	26±13
*z*	12.4±1.6	12.8±2.6	11.1±0.6*	7.0±1.1*	26.0±0.7	24.6±1.9	26.0±0.7	24.7±2.0	8.1±0.7*	5.8±1.7*	18.0±1.2	18.9±2.9

Note that values are means ± S.D.

*Statistically significant gait effects are indicated by an asterisk (P<0.05).

**Fig 7:**
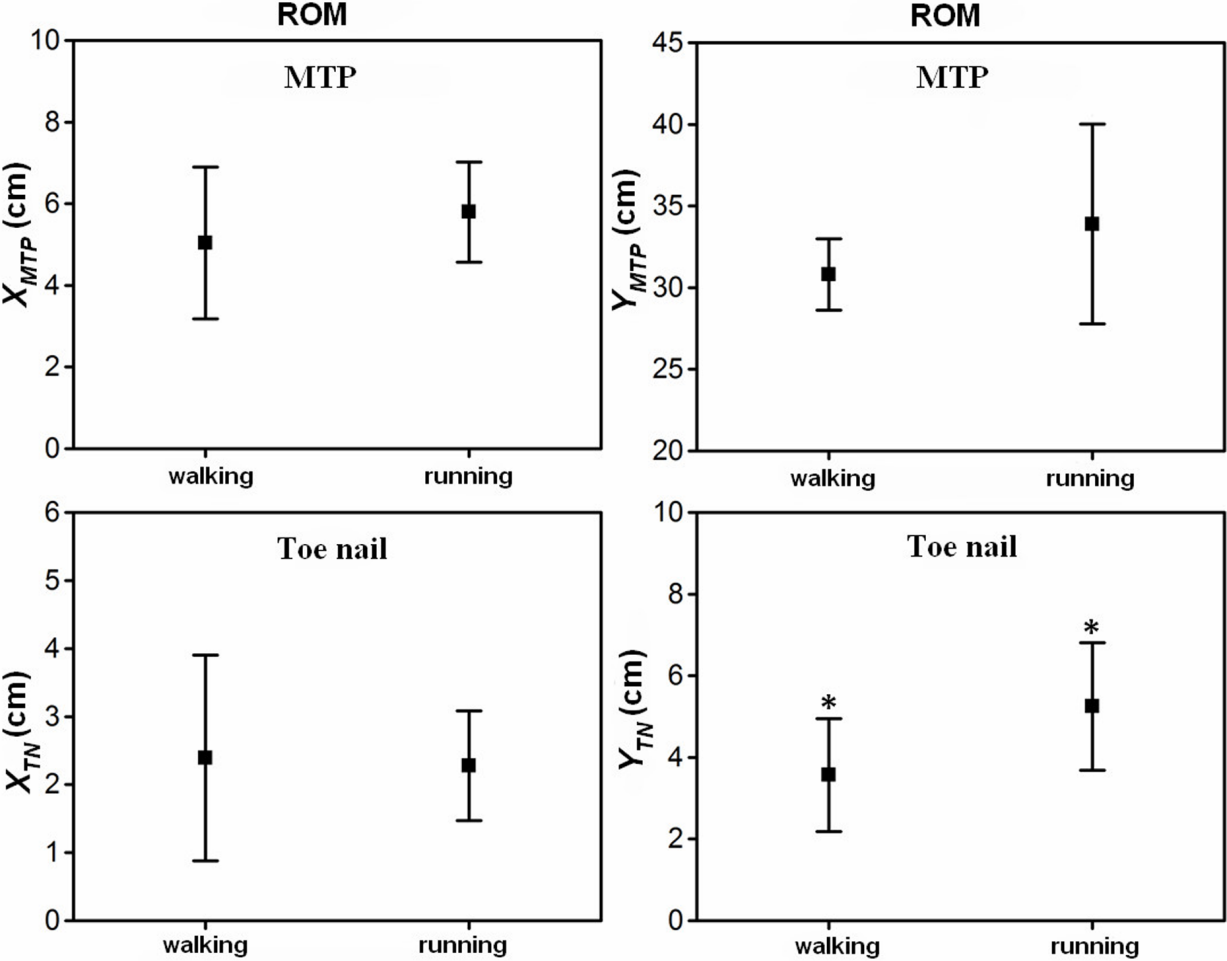
The averages and standard deviations of the lateral and forward displacements of the metatarsophalangeal joint and toenail at the ranges of motion during walking and running. A statistically significant effect of gait pattern is indicated by an asterisk (P < 0.05).

The results of the statistical analysis examining the effect of gait pattern on the key indicator (displacements at range of motion) of the lateral and forward displacements of the metatarsophalangeal joint and toenail are listed in Fig 7 and S3 Table. Gait pattern had no significant effect on the range of motion of the lateral and forward displacement of the metatarsophalangeal joint, whereas there was a great difference between the forward displacement *Y*_*MTP*_ (32 cm) and the lateral displacement *X*_*MTP*_ (5.0 cm). In addition, no statistically significant difference was found for the range of motion of the lateral displacement of the toenail (*X*_*TN*_). The gait pattern had a marked effect on the range of motion of the forward displacement of the toenail (*Y*_*TN*_). In walking and running, the ranges of motion of forward displacement of the toenail were 3.6 cm and 5.2 cm, respectively.

### Toe joint kinematics between solid ground and loose sand substrate

**Fig 8:**
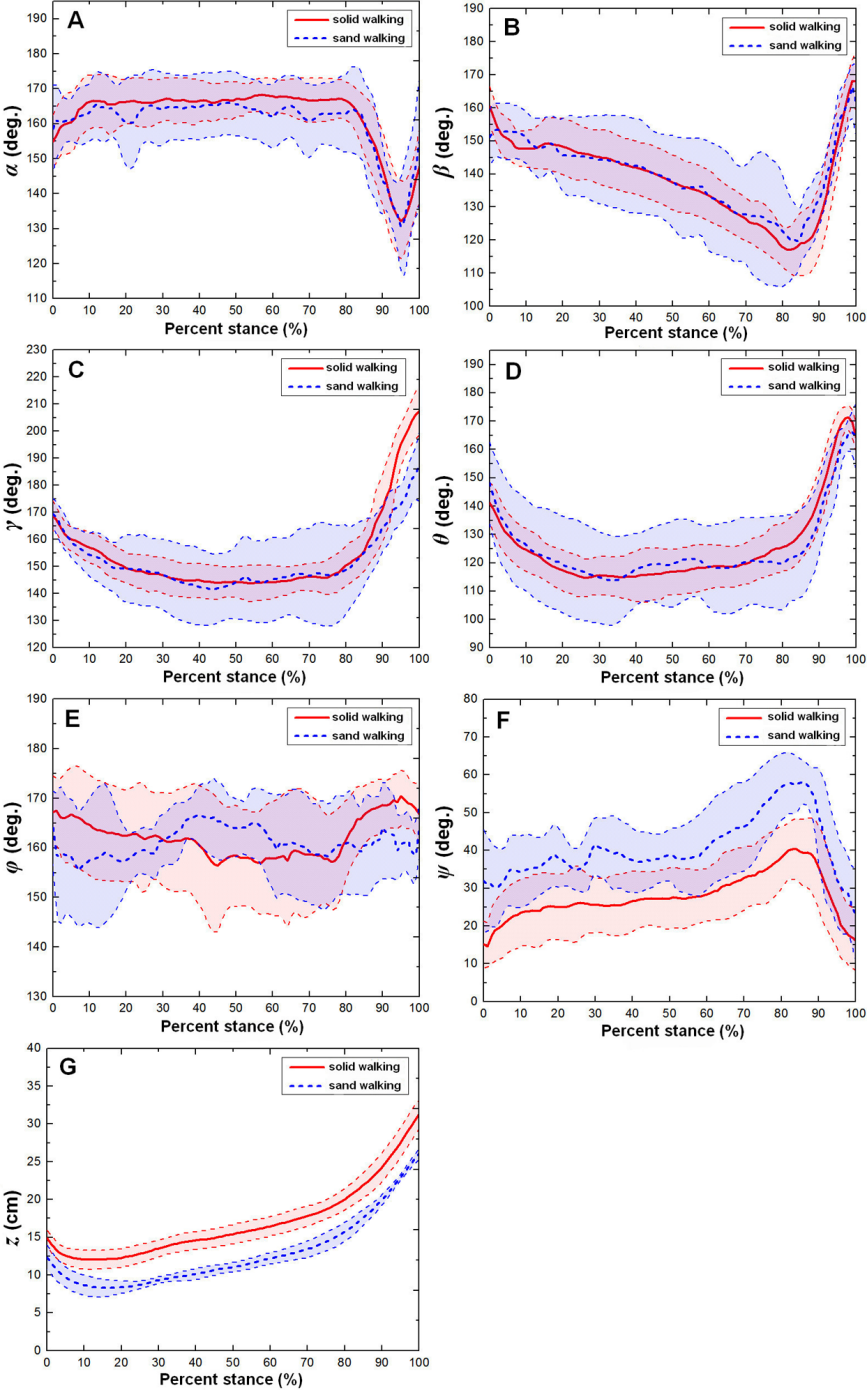
The averages and one standard deviations of the six toe joint angles and the vertical displacement of the metatarsophalangeal joint (*α*, *β*, *γ*, *θ*, *φ*, *ψ*, *z*). **They correspond to A, B, C, D, E, F, and G over the stance phases for all walking trials on the sand surface (blue dotted line)** and solid surface (red solid line). The angle decrease represents flexion, while the angle increase indicates extension.

Fig 8 shows the averages and one standard deviations of the six toe joint angles and the vertical displacement of the metatarsophalangeal joint (*α*, *β*, *γ*, *θ*, *φ*, *ψ*, *z*) over the stance phases for all walking trials on loose sand and solid substrate (see S2 and S4 Tables) [9]. In the walking gait, toe interphalangeal joint angles (*α*, *β*), the MTP3 joint angle (*γ*) and the MTP4 joint angle (*θ*) presented very similar motion patterns on loose sand and solid substrate. The first phalangeal joint angle of the 4th toe (*φ*) showed the largest motion variability among all six toe joint angles and no clear patterns during the whole stance phase. However, on the loose sand substrate, the joint angle (*ψ*) between the first phalanges of the 3rd and 4th toes was larger than that on the solid ground. This suggested that on loose, deformable substrates, ostriches may need to largely adjust the relative motion between the 3rd and 4th toes to maintain balance. In addition, during the stance phase, the metatarsophalangeal joint was closer to the substrate from solid ground to the loose sand surface. This may be mainly caused by the sinkage of the ostrich foot in locomotion.

**Fig 9:**
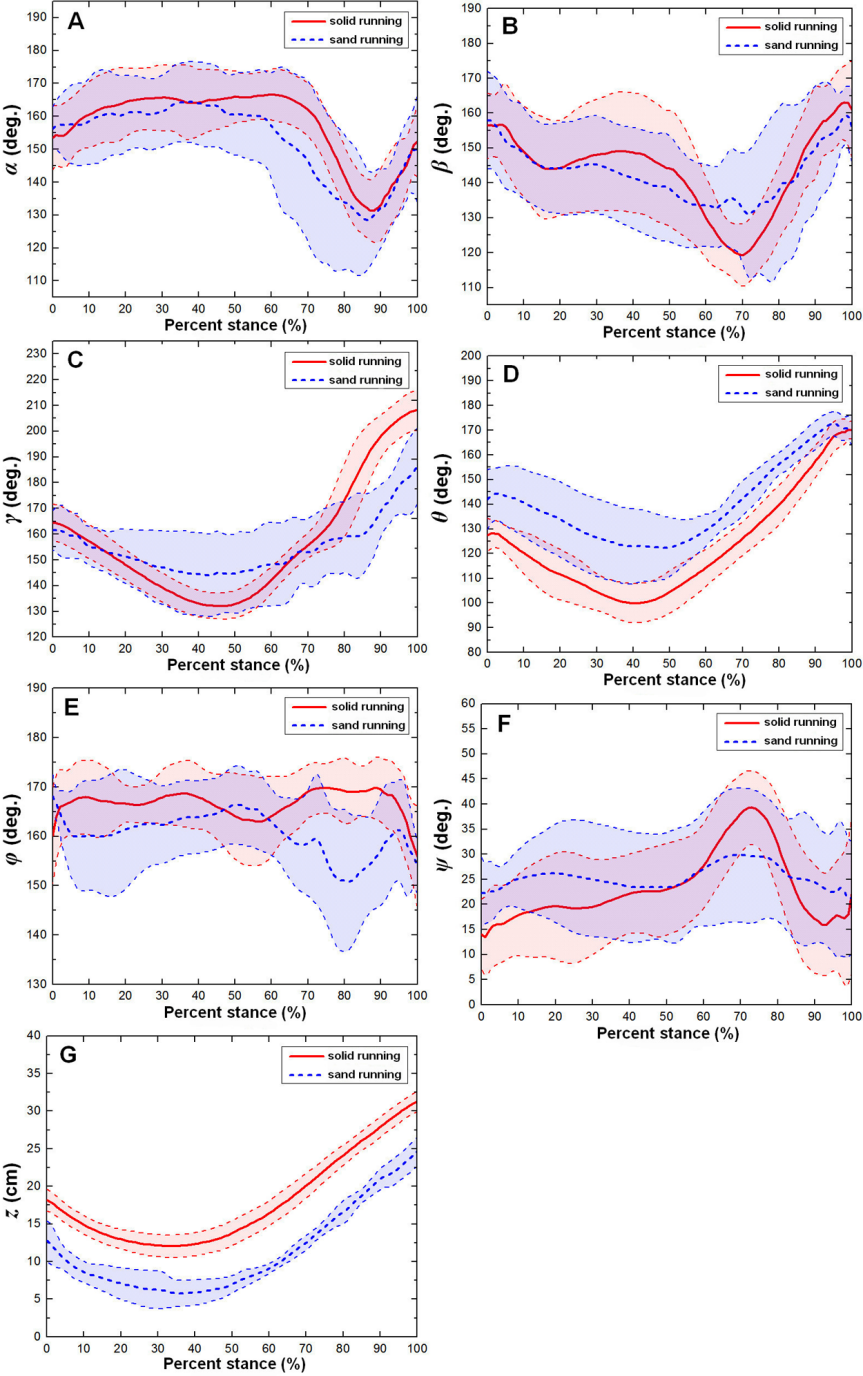
The averages and one standard deviations of the six toe joint angles and the vertical displacement of the metatarsophalangeal joint (*α*, *β*, *γ*, *θ*, *φ*, *ψ*, *z*). They correspond to A, B, C, D, E, F, and G over the stance phases for all running trials on the sand surface (blue dotted line) and solid surface (red solid line). The angle decrease represents flexion, while the angle increase indicates extension.

Fig 9 shows the averages and one standard deviations of the six toe joint angles and the vertical displacement of the metatarsophalangeal joint (*α*, *β*, *γ*, *θ*, *φ*, *ψ*, *z*) over the stance phases for all running trials on the sand surface and solid surface. In the running gait, toe interphalangeal joint angles (*α*, *β*), the MTP4 joint angle (*θ*) and the vertical displacement of the metatarsophalangeal joint (*z*) presented very similar motion patterns during the stance phase on loose sand and solid substrate. In contrast, on solid ground, the second phalangeal joint of the 3rd toe presented larger motion variability than that on loose sand substrate, which suggested that loose sand had an effect on rescuing impact force in ostrich locomotion. The phrase "rescuing impact force" means that the sand dampens the impact from the ground surface and dissipates energy. On the loose sand substrate, the MTP4 joint angle (*θ*) was greater than that on the solid ground. The first phalangeal joint angle of the 4th toe (*φ*) showed the largest motion variability among all six toe joint angles and no clear patterns during the whole stance phase. The metatarsophalangeal joint was closer to the substrate on the loose sand surface than on the solid ground.

However, the MTP3 joint angle (*γ*) and the joint angle (*ψ*)between the first phalanges of the 3rd and 4th toes had different motion patterns during the stance phase on solid ground and loose sand substrate in running (see Fig 9C and 9F). On solid ground, the MTP3 joint angle (*γ*) decreased by approximately 30 degrees (from touch-down to mid-stance) and then increased from approximately 135 degrees to 205 degrees before lift-off. On the loose sand substrate, the MTP3 joint angle (*γ*) decreased from approximately 160 degrees to 145 degrees (from touch-down to 40% of the stance phase), thereafter remained stable until reaching 60% of the stance phase and then increased slightly to 190 degrees before lift-off (see Fig 9C).

On solid ground, the joint angle (*ψ*) between the first phalanges of the 3rd and 4th toes increased first at touch-down and then decreased at 70% of the stance phase (see Fig 9F). On the loose sand substrate, the joint angle (*ψ*) fluctuated approximately 25 degrees during the whole stance phase. Therefore, on solid ground, the 4th toe abduction to the 3rd toe main axis was larger than that on loose sand substrate.

### Effect of ground medium on key indicators of toe joint angles and displacements

**Fig 10:**
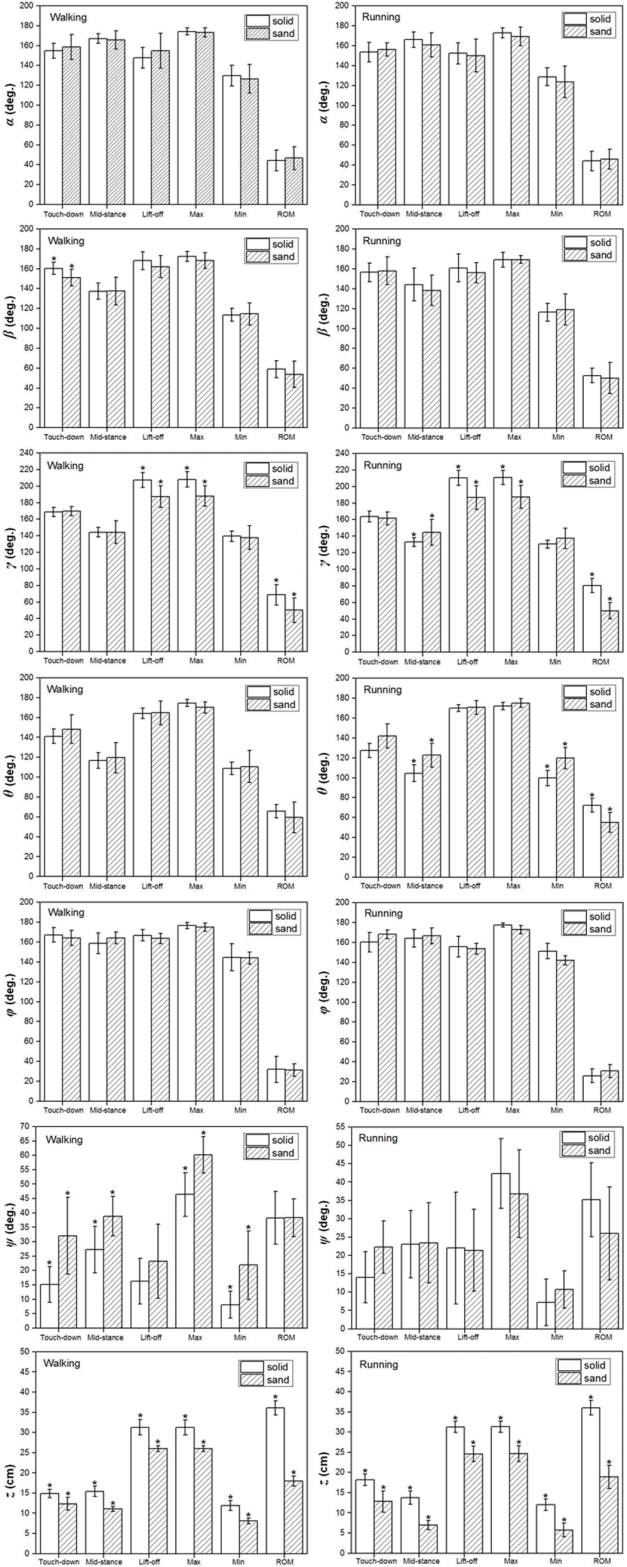
The averages and standard deviations of the six toe joint angles and the vertical displacement of the metatarsophalangeal joint at touch-down, mid-stance, lift-off, maximum, minimum and also the ranges of motion during walking and running between solid ground and sand surface. Statistically significant effects of ground medium differences are indicated by an asterisk (P < 0.05).

Fig 10 and S2 Table show the statistically significant effect of the ground medium difference between the solid and sand substrates on the six toe joint angles and the vertical displacement of the metatarsophalangeal joint at touch-down, mid-stance, lift-off, maximum, minimum and also the ranges of motion during the walking and running gaits. In walking, the MTP3 joint had the largest range of motion, and the first phalangeal joint of the 4th toe had the smallest range of motion among the six joint angles. Ground medium had a significant effect on the lift-off angle, the angle maximum and the range of motion of the MTP3 joint angle (*γ*). Statistically significant differences were found for the touch-down angle of the second phalangeal joint angle (*β*), the touch-down angle, mid-stance angle, the maximum and minimum of the joint angle (*ψ*) between the first phalanges of the 3rd and 4th toes. However, ground medium had no marked effect on the six key indicators of the phalangeal joint angles (*α*, *θ*, *φ*) between solid ground and loose sand substrate. On loose sand substrate, the second phalangeal joint of the 3rd toe flexed more at touch-down than on solid ground. Additionally, on a loose sand substrate, the joint angle (*ψ*) between the first phalanges of the 3rd and 4th toes at touch-down and mid-stance was larger than that on solid ground.

In running, no statistically significant differences were found for the six key indicators of the MTP3 joint angle (*γ*) and the MTP4 joint angle (*θ*) between solid ground and loose sand substrate. On the solid ground, the joint angles (*γ*, *θ*) at mid-stance and the range of motion were greater than those on the loose sand substrate. However, ground medium had no statistically significant effect on the six key indicators of the joint angles (*α*, *β*, *φ*, *ψ*).

In the walking and running gaits, the ground medium effect was found for the six key indicators (angles/displacements at touch-down, mid-stance, lift-off, maximum, minimum and also the ranges of motion) of the vertical displacement of the metatarsophalangeal joint (*z*). The range of motion of the vertical displacement (*z*) was larger on solid ground than on loose sand substrate.

### Sand particle velocity field and force field

**Fig 11:**
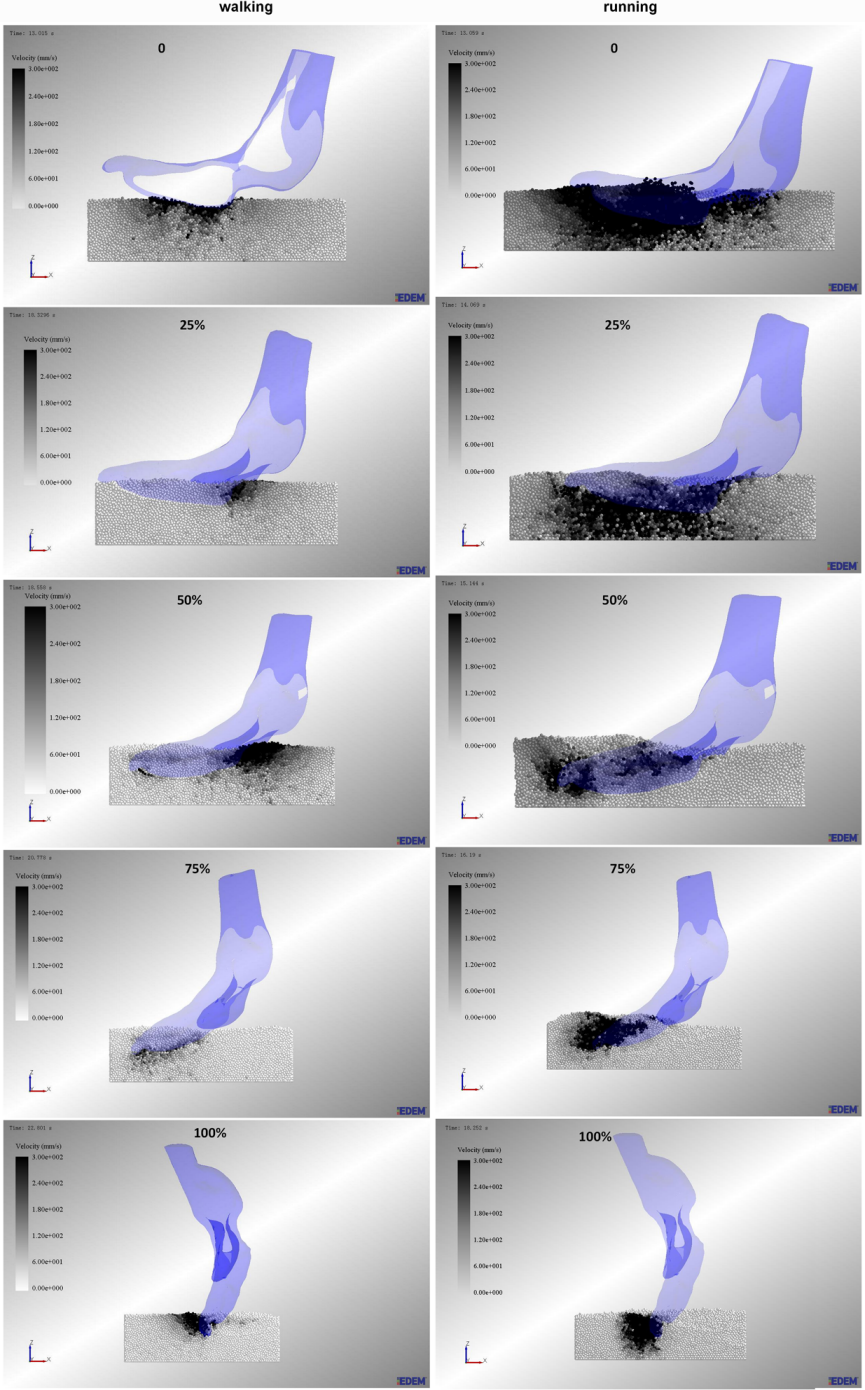
Particle velocity field under the ostrich toe plantar surface. In walking and running, the particle velocity field of five toe postures (0 posture, 25% posture, 50% posture, 75% posture, 100% posture) during the stance phase.

Fig 11 shows the walking and running velocity field of sand particles under five ostrich toe postures (0 posture, 25% posture, 50% posture, 75% posture, 100% posture) during the stance phase. The particle velocity varied from small to large, and the corresponding color also became deeper (varied from white to black), with a speed range of 0 to 0.3 m/s. In the running gait, the velocity of sand particles under the ostrich foot was larger than that in the walking gait, which indicated that in running, the sand particles were more active. In the process of traveling on sand, the area with larger particle velocity for 0 posture in the walking gait was mainly concentrated in the contact area of foot/sand particle, and there was no obvious velocity in the deeper region. In running, the particle velocity field was widely distributed and spread as "fan-shaped". At the mid-stance of traveling on sand, for 25% posture and 50% posture, the area with larger particle velocity in walking was located under the metatarsophalangeal joint, and the particle velocity is very small below the foot plantar. However, the particle field in running was "V-Shape" distribution. At the lift-off of traveling on sand, the particle velocity field was clearly distributed around the toenail. As the locomotor speed increased, the sand substrate had an impact on the toe plantar at touching the sand, and the particle velocity was large. When the locomotor speed decreased, the squeezing effect of the toes on the sand was smaller, and the particles absorbed the ground reaction force from the ostrich toe, which led to the small particle velocity field.

**Fig 12:**
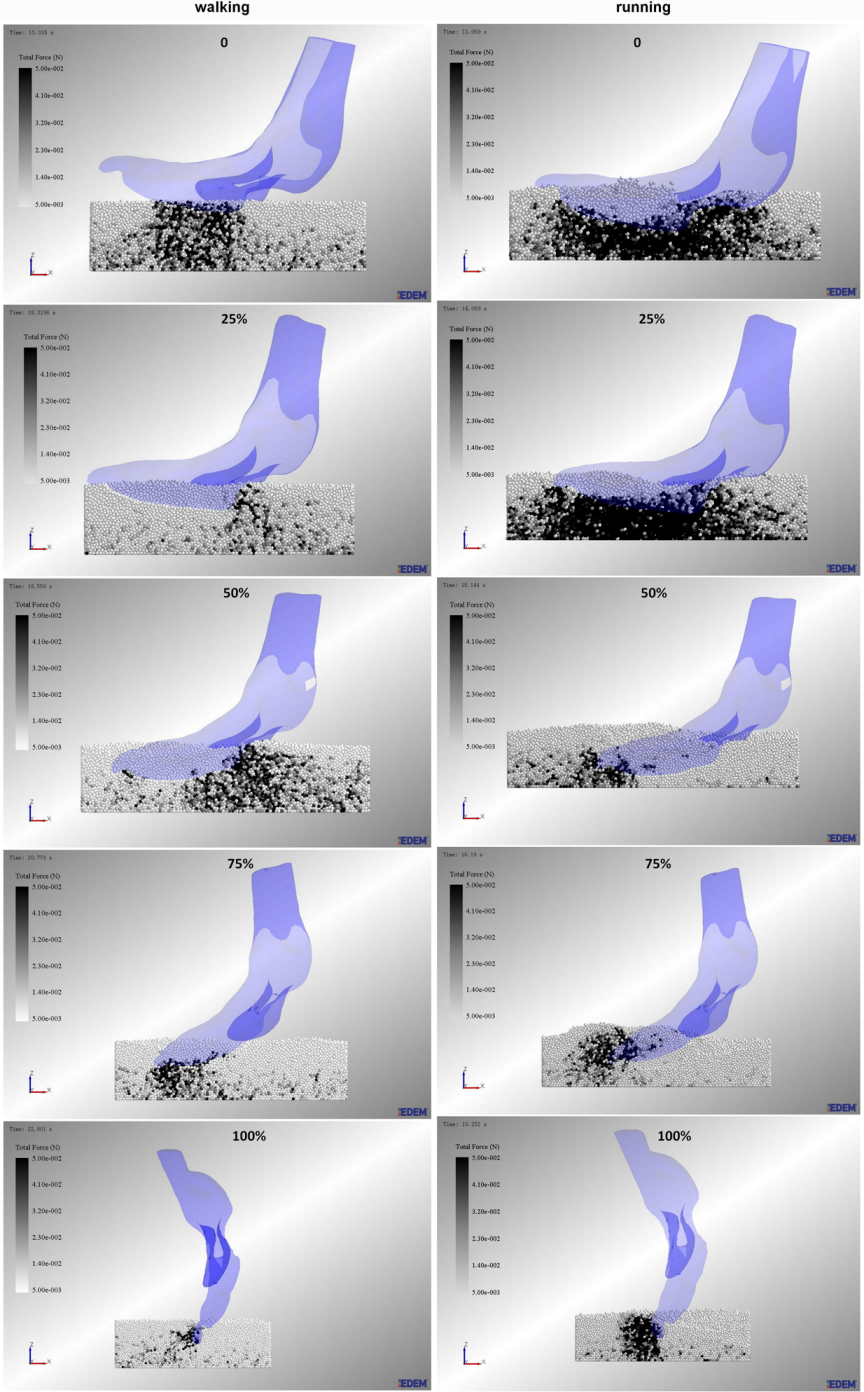
Particle force field under the ostrich toe plantar surface. In walking and running, the particle velocity field of five toe postures (0 posture, 25% posture, 50% posture, 75% posture, 100% posture) during the stance phase.

Fig 12 shows the walking and running force field of sand particles under five ostrich toe postures (0 posture, 25% posture, 50% posture, 75% posture, 100% posture) during the stance phase. During the whole stance period, the particle force field in the walking gait showed a transition from the foot plantar to the foot heel and then changed to the toenail. The particle force field in the running gait showed a direct change from the foot plantar surface to the toenail. Overall, in running, the particle force field was denser than in walking, and the phenomenon of particle extrusion was more obvious. From the touch-town to 20% of stance period, the force field of the 0 posture of walking was a "rectangular" shaped distribution and mainly concentrated in the second phalanx of the third toe. However, the distribution of the particle force field in the running gait was greater than that in the walking gait, which presented the tendency to encircle toes. From the 20% to 40% stance period, compared to the walking gait, the particle force field of 25% posture was more dense and uniform, which may maintain the body balance in locomotion. From 40% to 60% of the stance period, the particle force fields for 50% posture had a significant difference between walking and running. The areas with larger particle forces were located in the toe heel plantar surface and toenail. From 60% to 100% of stance period, compared to walking gait, the particle force field of 75% posture and 100% posture were more dense, which likely helped to effectively improve the grip and traction performance of the toe movement in a sandy environment. It should be noted that we have used running postures to simulate both walking and running processes, and this is due to that gait pattern had no significant effect on the key indicators of the toe joint angles on sand substrate.

### Particle disturbance in different depths

**Fig 13:**
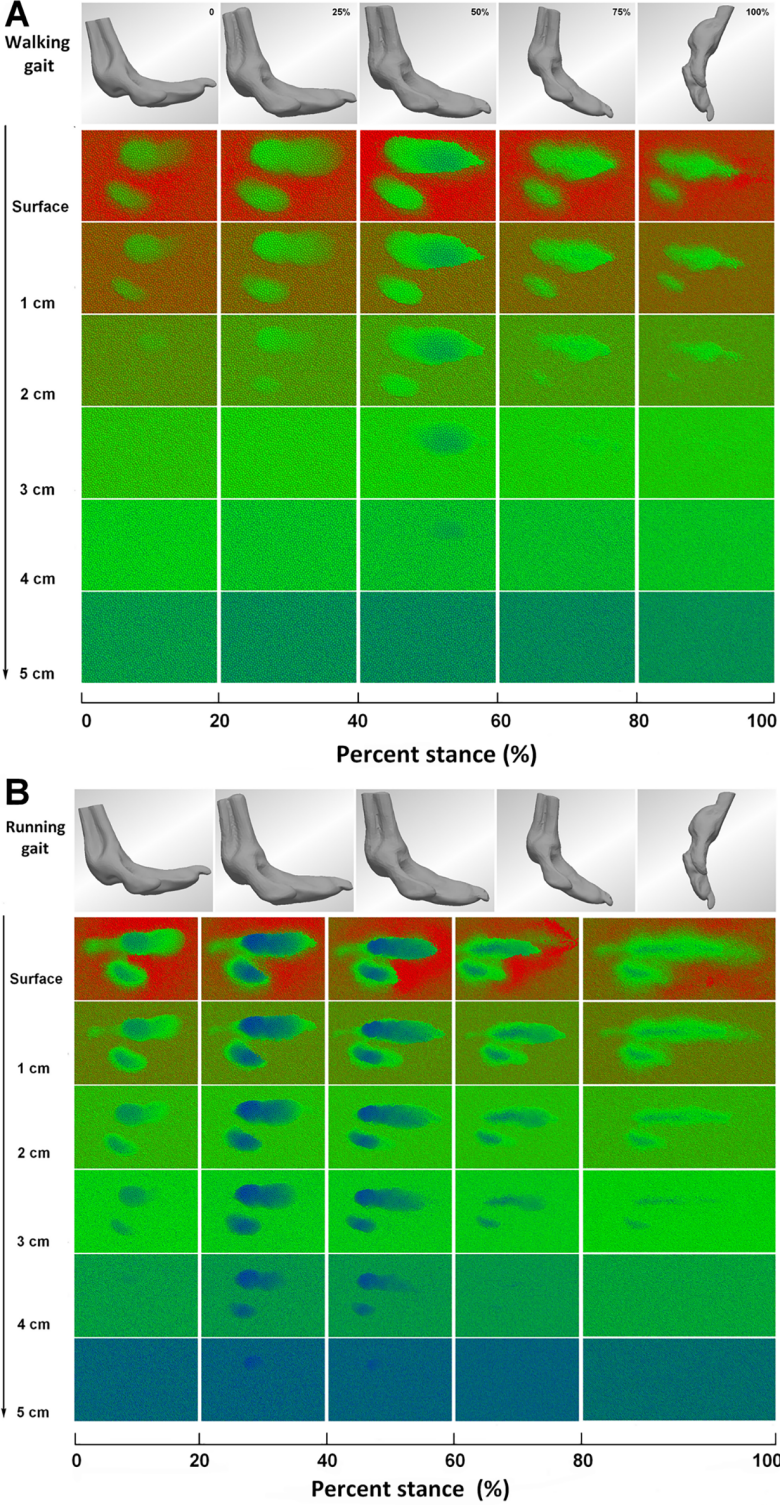
Ostrich toe footprints at different particle planes during the stance phase. A In walking gait. B In running gait.

Fig 13 shows the ostrich toe footprints at different particle planes during the stance phase in the walking and running gaits. Red represents the higher position of the particle, and blue represents the lower position of the particle. In walking gaits on the sand surface, the footprints were shallow at touch-down and presented clear toenail contours at mid-stance; compression of the surrounding sand caused it to flow to the toe bunker and create a fuzzy boundary around the footprint. During the early stance and late stance, when the sand depth was greater than 3 cm, toe postures (0 posture, 25% posture, 75% posture, 100% posture) had a small disturbance on the sand substrate, which meant that toe postures did not disturb the sand below depths of 3 cm. However, at mid-stance, when the sand depth was 4 cm, toe postures still disturbed the sand (Fig 13A). In running, sand disturbance of toe posture decreased with increased sand depth. Compared to walking gaits, toe posture had a great disturbance on the deeper sand plane (Fig 13B).

### Contact force analysis and distribution of toe postures while traveling on sand

**Fig 14:**
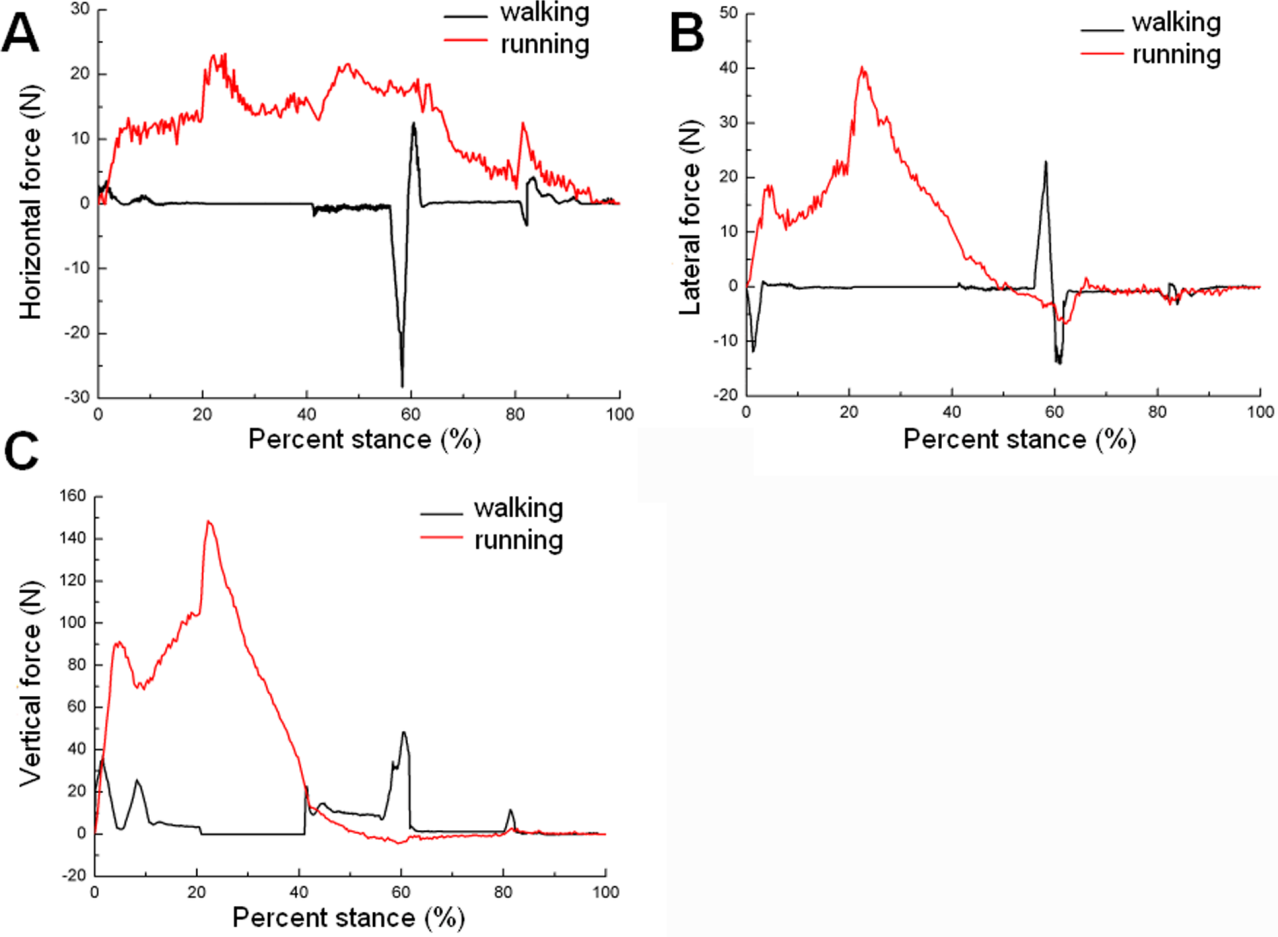
The force of sand particles on ostrich toe posture during the stance phase. A Horizontal force. B Lateral force. C Vertical force.

Fig 14 shows the force of the sand particles on the ostrich toe posture during the stance phase. In running, the horizontal force was larger than that in walking during the whole stance phase, which indicated that the sand surface needed to provide greater friction propulsion force with increased locomotor speed (see Fig 14A). From touch-down to 40% of the stance phase, the lateral force and vertical force were greater in running than walking, which indicated that 25% posture and 50% posture play an important role in anti-slip and anti-subsidence. From the 40% stance phase to lift-off, the lateral force and vertical force of the toe posture were very small. However, in walking at 60% of the stance phase, the lateral force and vertical force of toe posture appeared as peak values. This may be due to the strong compression of the sand by the toenail tip just before lift-off (see Fig 14B and 14C).

## Discussion

This research presents the first toe joint kinematic analysis of sub-adult ostriches for walking and running on loose sand substrates and dynamic simulations of ostrich toes traveling on sand. Reliable data on major toe joint angle trajectories, three-dimensional displacement of the metatarsophalangeal joint and displacement in the lateral, forward direction of the toenail were acquired based on a large number of trials. Because we chose two genetically unrelated subjects of the same sex with similarity in age and size, the consistency of inter-individual results in walking and running trials accurately document a generalized pattern in ostrich locomotion.

On loose sand substrates, ostrich gaits had significant differences in the stance phase, stride phase and stride length, as shown in Table 1. In the walking gait, the stance duration and stride duration were longer than that of the running gait, while the stride length in the running gait was larger than that of the walking gait. This indicated that the ostriches improve speed by increasing the stride length and step frequency and decreasing the stance duration. In addition, the swing duration had no significant differences between the walking and running gaits. Previous studies showed that the most notable differences in limb kinematics between birds and humans occur at the walk-run transition and are maintained as running speed increases. Changes in gait are smooth and difficult to discern in birds but distinct in humans [18]. Previous studies showed that the change of gait at the walk-run transition was smooth and difficult to discern in birds but distinct in humans, involving abrupt decreases in step length and duty factor (time of contact) and a corresponding increase in limb swing time. These differences appear to reflect a spring-like run that is stiff in humans (favoring elastic energy recovery) but more compliant in birds (increasing time of ground contact) [18]. The gait transition of ostriches was consistent with the above viewpoints.

In addition, we examined the differences in phalangeal joint kinematics in walking and running on solid ground [9] and loose sand substrates. For experiments of solid ground and loose sand substrates, the similarities are the experimental objects (two female ostriches), testing equipment (three synchronized high-speed cameras and three-dimensional motion tracking system) and the position of reflect markers on ostrich digits. The differences are the ground medium. In the loose sand substrate experiment, the runway was covered with a 3 mm non-slip rubber sheet to prevent potential damage from the ostrich foot. In the experiment of loose sand substrate, the runway was covered with 4 cm of loose sand particles.

### Similar toe postures while traveling on sand at slow and fast locomotion

The changes in ostrich toe posture came from the relative motion of the interphalangeal joints and the changes in joint angles. The first phalangeal joint angle of the 3rd toe (*α*) and the second phalangeal joint angle (*β*) represented similar motion patterns at the stance phases in the walking and running gaits. This showed that the interphalangeal joint kinematic of the 3rd toe was similar between slow walking and running. The MTP3 joint angle (*γ*) and the MTP4 joint angle (*θ*) revealed a noticeably stationary period in slow walking instead of the running gait. The first phalangeal joint of the 4th toe (*φ*) displayed the largest angle variability among all six toe joints. However, the angle (*ψ*) between the 3rd and 4th toes showed different patterns over the stance phase in the walking and running gaits.

The gait pattern had a significant effect on the maximum angle of the joint between the two toes during the stance phase. In walking, the maximum angle between the first phalanx of the 3rd and 4th toes was larger than that in running (see Table 3). This motion pattern may improve balance and stability of the body by adjusting the angle between the axis of the 3rd and 4th toes in the walking gait. Compared to the walking gait, the joint angle (*ψ*) between the two toes remained in a stable motion pattern and slightly fluctuated by approximately 25 degrees during running. In the fast running gait, the ostrich was in dynamic equilibrium, and the range of motion of the angle between the first phalanx of the 3rd and 4th toes was small. At slow locomotion, the clear motion pattern of the joint angle between the first phalanx of the 3rd and 4th toes suggested that the 4th toe had significant adduction and abduction relative to the 3rd toe. In the bipedal ostrich locomotion, the 4th toe provided a significant increase in the base of support because of its lateral orientation, resulting in the typical triangular pressure profile [4]. The interphalangeal ligament inserting mediodistally at the proximal phalanx of the 3rd toe and medioproximally at the second phalanx of the 4th toe regulated the coupled toe motion of the 3rd and 4th toes [4]. In addition, this ligament also limits maximum toe abduction to the 3rd toe main axis. Thus, the interphalangeal ligament inserted at the proximal phalanx of the 3rd toe and the second phalanx of the 4th toe may help ostriches maintain static balance in slow locomotion and dynamic balance in fast locomotion.

The MTP3 joint angle (*γ*) and the MTP4 joint angle (*θ*) presented very similar motion patterns between walking and running on loose sand, which showed that the motions of the MTP3 and MTP4 joints were highly synchronized. A previous study[9] showed that the motions of the MTP3 and MTP4 joints are highly synchronized across the entire speed range, and the 3rd and 4th toes actually work as an ‘‘integrated system” with the 3rd toe as the primary load bearing element with the 4th toe as the complementary load sharing element mainly to ensure the lateral stability of the permanently elevated metatarsophalangeal joint. Thus, on a loose sand substrate, the 3rd toe and 4th toe were coupled as a whole in locomotion.

According to the phalangeal joint kinematics and statistical analysis (see Fig 6 and Table 4), the gait pattern has no marked effects on the phalangeal joint angles of the two toes at touch-down, mid-stance, lift-off and the range of motion during the stance phase on loose sand substrate. This suggests that the key postures of ostrich toes are similar in the process of traveling on sand between slow walking and running. However, the key postures of ostrich toes were significantly different during the stance phase on solid ground [9]. Therefore, the ground medium becomes the key factor in the toe postures that ostriches adopted during the stance phase from slow to fast locomotion.

The ostrich toes insert and shear substrate of loose sand and may lose energy just like that the hind foot of the zebra-tailed lizard rotated and paddled through fluid granular medium. The upper hind leg muscles of lizard performed three times as much mechanical work on the granular surface as on the solid surface to compensate for the greater energy lost within the foot and to the substrate [12–13]. As animals move across natural surfaces, energy is dissipated both within their body and limbs and to the environment [19–20]. The multi-jointed muscle-tendon system in ostriches functionally interconnects pelvis and toes and couples the flexion and extension of joints throughout the hindlimb [21]. Therefore, ostriches may adjust the phalangeal joint locomotion of two toes by using elastic elements such as tendons and ligaments that can function as springs to store and return energy during rapid locomotion, such as running to decrease energetic cost [1]. On the other hand, the change of gait at the walk-run transition in ostrich is smooth, which may cause no significant differences in the interphalangeal joint angles of two toes from the walking to running gaits. Finally, some researchers observed that the ostrich foot has four toe-pads and three cushions, which are likely to be responsible for the protection of the underlying soft tissues and absorption of concussion [22]. The unique modified structures of ostrich foot, such as toe-pads and cushions, may also be adapted for long-distance movement in desert land and sandy environments.

### The anti-slip ability of ostrich toenails and the motion of the metatarsophalangeal joint

In walking, the lateral displacement (*X*_*TN*_) of the metatarsophalangeal joint increased by approximately 1.0 cm from touch-down to 20% of the stance phase and then remained stable before lift-off. However, in running, the lateral displacement (*X*_*TN*_) of the toenail presented no apparent pattern. In addition, no statistically significant difference was found for the range of motion of the lateral displacement of the toenail (*X*_*TN*_). The gait pattern had only a marked effect on the range of motion of the forward displacement of the toenail (*Y*_*TN*_). In walking and running, the range of motion of the forward displacement of the toenail was 3.6 cm and 5.2 cm, respectively. In walking trials, the claw exerted only minor pressure throughout the stance phase, while at running speeds, the claw exerted 5 times the amount of pressure observed in walking [4]. At the same time, in the toe posture dynamics simulation of traveling on sand, the particle velocity and force fields around the toenail were larger and denser in running than that in slow walking. Additionally, because the pads of the plantar surfaces would not provide sufficient traction at speeds of 16 m/s, especially on granular surfaces, the claw provides a solid positional anchor once embedded in the loose sand substrate [4]. Therefore, the ostrich toenail plays an important anti-slip role and provides a traction force at push-off on the sandy environment in fast locomotion.

The range of motion of the vertical displacement (*z*) was larger on solid ground than on loose sand substrate. Statistically significant differences were also found for the mid-stance displacement of the vertical displacement of the metatarsophalangeal joint (*z*). Therefore, compared to running on loose sand substrate, the metatarsophalangeal joint was at a statistically higher position at mid-stance during walking, which was consistent with the viewpoints that on solid ground, the metatarsophalangeal joint was positioned closer to the ground as speed increased. The MTP joint had a greater range of motion during running than that in slow walking, which may further explain the metatarsophalangeal joint being considered as energy storage and shock absorption during fast locomotion[9]. This showed that the metatarsophalangeal joint played an important role in energy saving in fast locomotion on loose sand substrate.

In summary, first, the key postures of ostrich toes are similar in the process of traveling on sand between slow walking and running. However, the key postures of ostrich toes were significantly different during the stance phase on solid ground. Therefore, the ground medium is the key factor in the toe postures that ostriches adopted during the stance phase from slow to fast locomotion. Secondly, because the 3rd toe and the 4th toe are connected by the interphalangeal ligament, the motions of the MTP3 and MTP4 joints were highly synchronized; on loose sand substrate, the 3rd toe and 4th toe were coupled as a whole with the 3rd toe as the primary load bearing element with the 4th toe as the complementary load-sharing element to mainly ensure the lateral stability of the permanently elevated metatarsophalangeal joint. Thirdly, the ostrich toenail plays an important anti-slip role and provides a traction force at push-off on the sandy environment in fast locomotion. The metatarsophalangeal joint played an important role as the energy saving in fast locomotion on loose sand substrate.

## Conclusion

The three-dimensional motion track analysis system Simi Motion shows that gait pattern has no significant effect on the key indicators (angles at touch-down, mid-stance, lift-off and range of motion) of the toe joint angles (*α*, *β*, *γ*, *θ*, *φ*, *ψ*) and that ostrich toe phalanges motion postures are basically the same. The ground medium is the key factor in the toe postures that ostriches adopted during the stance phase from slow to fast locomotion. The 3rd toe and the 4th toe are connected by the interphalangeal ligament, and the motions of the MTP3 and MTP4 joints were highly synchronized; on loose sand substrate, the 3rd toe and 4th toe were coupled as a whole to maintain static balance in slow locomotion and dynamic balance in fast locomotion. In addition, the gait pattern had a marked effect on the range of motion of the forward displacement of the toenail (*Y*_*TN*_). The ostrich toenail plays an important anti-slip role and provides a traction force at push-off on the sandy environment in fast locomotion. The metatarsophalangeal joint played an important role in energy saving in fast locomotion on loose sand substrate. Simulations reveal that the particle velocity field, particle force field and sand particle disturbance in the running gait are denser than those in the walking gait.

## Supporting information

S1 Table Simulation motion data. The velocity and acceleration in the process of the ostrich foot traveling on sand during the stance phase in the walking and running gaits.
(XLS)

S2 Table Statistical analysis of the key indicators of phalangeal joint angles and displacements. On loose sand substrate, the statistical analysis results of gait pattern on key indicators. In the walking gait, the statistical analysis results of ground medium on key indicators. In the running gait, the statistical analysis results of ground medium on key indicators.
(XLS)

S3 Table Statistical analysis of the displacements of the MTP and toenail at different ranges of motion. On the loose sand substrate, the statistical analysis results of gait pattern on the indicator of the displacements.
(XLS)

S4 Table The averages and standard deviations of the toe joint angles and displacements on loose sand substrate and solid ground during walking and running. The toe joint angles *α*, *β*, *γ*, *θ*, *φ* and *ψ*. The vertical displacement of the metatarsophalangeal joint *z*. The lateral (X-direction) and forward (Y-direction) displacements of the metatarsophalangeal joint and toenail (*XMTP*, *YMTP*, *XTN*, *YTN*).
(XLS)
